# Some new insights into the biological activities of carboxymethylated polysaccharides from *Lasiodiplodia theobromae*

**DOI:** 10.1186/s12906-023-04190-7

**Published:** 2023-10-07

**Authors:** Matheus Cerdeira Pires, Natalia de Gois Andriolo, Bruno Rafael Pereira Lopes, Ana Lucia Tasca Gois Ruiz, Valeria Marta Gomes do Nascimento, Karina Alves Toledo, Catarina dos Santos

**Affiliations:** 1 Experiential Master of Science in Biotechnology, College of Science, Northeastern University, Boston, MA USA; 2 LAQUA (Laboratório de Química da Unesp Assis), University of São Paulo State (UNESP), Assis, SP Brazil; 3 Continuing Education Program in Economics and Business Management (PECEGE), Superior School of Agriculture “Luiz de Queiroz” University of São Paulo (USP) (Esalq-USP), Piracicaba, São Paulo, Brazil; 4 Laboratory of Cellular and Molecular Immunology, University of São Paulo State (UNESP), Assis, SP Brazil; 5 Farmacologia e Toxicologia Experimental), LAFTEx (Laboratório de Fitoquímica, State University of Campinas, Campinas, SP Brazil; 6 Laboratory of Biochemistry and Bioprocess, University of São Paulo State (UNESP), Assis, SP Brazil

**Keywords:** Lasiodiplodan, Immunomodulatory and Antiviral, Antiproliferative, Anticoagulant activities

## Abstract

**Background:**

Carboxymethylated Lasiodiplodan (LaEPS-C), *Lasiodiplodia theobromae* β-glucan exopolysaccharide derivative, has a well-known range of biological activities. Compared to LaEPS-C, its fractions, Linear (LLaEPS-C) and Branched (BLaEPS-C), have biological potentialities scarcely described in the literature. So, in this study, we investigate the immunomodulatory, antiviral, antiproliferative, and anticoagulant activities of LLaEPS-C and BLaEPS-C and compare them to the LaEPS-C.

**Methods:**

LaEPS was obtained from *L. theobromae* MMBJ. After carboxymethylation, LaEPS-C structural characteristics were confirmed by Elementary Composition Analysis by Energy Dispersive X-Ray Detector (EDS), Fourier Transform Infrared (FTIR), and Nuclear Magnetic Resonance (NMR). The immunomodulatory activity on cytokine secretion was evaluated in human monocyte-derived macrophage cultures. The antiviral activity was evaluated by Hep-2 cell viability in the presence or absence of hRSV (human respiratory syncytial virus). In vitro antiproliferative activity was tested by sulforhodamine B assay. The anticoagulant activity was determined by APTT (Activated Partial Thromboplastin Time) and PT (Prothrombin Time).

**Results:**

LaEPS-C showed low macrophage cell viability only at 100 µg/mL (52.84 ± 24.06, 48 h), and LLaEPS-C presented no effect. Conversely, BLaEPS-C showed cytotoxicity from 25 to 100 µg/mL (44.36 ± 20.16, 40.64 ± 25.55, 33.87 ± 25.16; 48 h). LaEPS-C and LLaEPS-C showed anti-inflammatory activity. LaEPS-C presented this at 100 µg/mL (36.75 ± 5.53, 48 h) for IL-10, and LLaEPS-C reduces TNF-α cytokine productions at 100 µg/mL (18.27 ± 5.80, 48 h). LLaEPS-C showed an anti-hRSV activity (0.7 µg/ml) plus a low cytotoxic activity for Hep-2 cells (1.4 µg/ml). LaEPS-C presented an antiproliferative activity for NCI-ADR/RES (GI_50_ 65.3 µg/mL). A better PT was achieved for LLaEPS-C at 5.0 µg/mL (11.85 ± 0.87s).

**Conclusions:**

These findings demonstrated that carboxymethylation effectively improves the biological potential of the LaEPS-C and their fractions. From those polysaccharides tested, LLaEPS provided the best results with low toxicity for anti-inflammatory, antiviral, and anticoagulant activities.

## Background

*Lasiodiplodia theobromae* MMPI (Botryosphaeriaceae) is a filamentous fungus and a non-host-specific plant pathogen that has a great incidence in tropic and subtropic areas. It has been associated with plant diseases such as fruit and root rot, dieback, and canker, leading to significant economic loss in commodities such as mango, banana, citrus, etc. It affects plants in various stages, including postharvest time [1, 2]. It has also been described as an agent of infections such as sinusitis, keratitis, pneumonia, and cutaneous lesions in immunocompetent and immunocompromised human patients [2].

Besides its pathogenicity, *L. theobromae* MMPI has been catching the interest of researchers because of the biosynthesis of Lasiodiplodan, an β-glucan [1–3] exopolysaccharide. Exopolysaccharides (EPS) can be found attached to the cell surface or in the fungal extracellular medium as slime. They promote enhancement in the interactions between the fungus and its host and protect it against dissection, virus, and protozoan attacks [4, 5]. In the case of *L. theobromae*, the EPS produced is composed of three types of β-glucans: a branched chain with β-(1→3)(1→6) type bonds, representing a proportion of 67% (m/m) of the polysaccharide, and two linear chains with β-(1→6) type bonds, but with different molecular masses (7,000 g/mol and 1,800,000 g/mol) [6]. Also, they differ in conformation, physical properties, binding affinity to receptors, and, thus, biological functions [7–9].

The β-glucans like LaEPS are PAMPs (Pathogen-Associated Molecular Patterns), which trigger innate immunity mechanisms [10]. PAMPs recognition is made from PRRs (Pattern Recognition Receptors), molecules that function as receptors on the surface or inside immune cells [11]. Among the PRR receptors, Dectin-1 is a receptor that recognizes polysaccharides containing β-(1→3) and/or β-(1→6) bonds and from the β-(1→6) branches [12]. Therefore, the *L. theobromae* EPS (LaEPS) comprises glucans that Dectin-1 can recognize [13].

The glucan-receptor interaction generates the activation of the immune cell, producing three possible cellular responses: phagocytic activity, inflammatory mediators, and production of cytokines, such as IL-10 and TNF-α [12, 14]. TNF-α and IL-10 have opposing roles in the immune system. While TNF-α is a pro-inflammatory cytokine capable of promoting the activation of neutrophils and vessel endothelial cells, IL-10 is an anti-inflammatory cytokine whose main action includes the negative regulation of the immune system from inhibiting the synthesis of pro-inflammatory cytokines [15, 16]. Some authors suggest that triple helical structure conformation and the presence of hydrophilic groups are possibly crucial for immune activity and anti-cancer effects [9, 17–19].

LaEPS has been reported as having an antiproliferative effect on breast cancer cells, hypoglycemic function, antimicrobial and antioxidant activities, making it an attractive biopolymer for pharmaceutical, food, and cosmetic applications [3, 17, 20, 21]. Moreover, Lasiodiplodan has an easier production compared to the other exopolysaccharides since its fermentation is submerged, and its recovery is made by precipitation methods from the cell-free fermentation broth in ethanol. So, research efforts have focused on improving its production, chemical derivatization, and biological activity analyses [20].

But, β-glucans are poorly soluble in water, their topical administration has low toxicity, and their intravenous administration is associated with hepatosplenomegaly, granuloma formation, and microembolization [13, 22]. This reduced water solubility is due to their triple helix structure, formed by the interaction of polyhydroxy groups and the outside surface of the chain, which restricts their physiological function in vivo [23].

One way to improve the solubility and enhance the biological activity of β-glucans is the production of derivatives *via* organic syntheses, such as carboxymethylation, sulphation, and acetylation [24]. Compared to non-carboxymethylated β-glucans, carboxymethylation of β-(1→6)-glucan increases antioxidant activity [8]. For β-(1→3)-glucan, carboxymethylation has been described as improving the antitumor activity [25]. The reduction of oxidative damage in healthy men through a carboxymethylated β-(1→3)(1→6)-glucan was also described [26].

Polysaccharides have also been described for their inhibitory activity against viruses, such as hRSV [27]. HRSV is an infectious agent in infants and young children with no vaccine. Commercial drugs such as Ribavirin had therapeutic effects on HRSV have been questioned. Moreover, Palivizumab (Synagis), another commercial drug, is high-cost [28]. So, cost-effective agents to prevent and manage HRSV infection are still needed. LaEPS-C or their fractions could be an alternative to the antiviral activity for hRSV (human Respiratory Syncytial Virus). They inhibit the first step of infection, where the glycoprotein on the viral envelope utilizes its positive charges to interact with negative charges of heparan sulfate (HS), one of the host cell surface receptors. By inhibiting this step, polysaccharides mimic HS, thus blocking the virus from entering the host cell [29, 30].

Sulfated β-glucans exert anticoagulant activity because of their negative charge. They prolonged the APPT concentration-dependent manner and showed values > 100 s for 20 µg/mL [31]. APTT (Activated partial thromboplastin time) and PT (Prothrombin time) are tools for diagnosing and monitoring coagulation disorders. The APTT measures the coagulation activity of the intrinsic pathway (coagulation factors II, V, VIII, IX, X, XI, and XII) and common pathways. PT is employed to evaluate the extrinsic and common pathways of coagulation, which would detect deficiencies of factors II, V, VII, and X and low fibrinogen concentrations. For instance, the PT range for healthy donors is between 11 and 13.5, and the time range of APTT is between 25 and 32 s [32, 33].

Despite the good activity presented by sulfated β-glucans, the conventional sulfation method has high costs and generates toxic waste due to the use of chlorosulfonic acid and organic solvents, such as pyridine [34]. Conversely, carboxymethylation possesses advantages, such as low-cost reagents, safe and low-toxic reaction products [35]. So, carboxymethylation should also be an alternative to sulfation since it may promote electronegativity similar to that found in heparin [36]. So, adding negative charges on the β-glucans by carboxymethylation gives this polysaccharide a heparinoid characteristic [8]. However, sulfation uses chlorosulfonic acid, which is unsafe despite being a cheap reagent. Carboxymethylation could be an alternative to sulphation, as the monochloroacetic acid is less dangerous and easier to obtain.

There are few studies comparing carboxymethylated β-glucan from *L. theobromae* (LaEPS-C) biological potentialities with their fractions LLaEPS-C (Linear carboxymethylated β-glucan *L. theobromae*) and BLaEPS-C (Branched carboxymethylated β-glucan from *L. theobromae*). Therefore, in this manuscript, we search to establish who is more effective by analyzing the modulating potential of the inflammation, antiviral, antiproliferative, and anticoagulant activities of those β-glucans and correlating them.

## Methods

### Microorganism cultivation, extraction, and purification of exopolysaccharides fractions

*L. theobromae* MMBJ was maintained at 4^o^C on potato-dextrose-agar. The Inoculum was prepared by growing *L. theobromae* MMBJ on agar plates containing Vogel’s medium [37], agar (20 g/L), and glucose (10 g/L) at 28 °C. After seven days, two agar plugs (0.5 cm diameter) containing fungal-colonized mycelium were taken and transferred to Erlenmeyer flasks (125 mL) containing 25 mL Vogel’s medium and glucose (0.5 g/L) and left at 28^o^C for 48 h on a rotary shaker (180 rpm). The pre-cultures were then homogenized (sterilized chilled Blender) for 0.5 min at maximum speed. Twelve mL aliquots of the mycelial homogenate were used to inoculate Erlenmeyer flasks (3 L) containing 600 mL of nutrient media comprising Vogel’s medium and sucrose (50 g/L). Cultures were grown in submerged cultivation (180 rpm, 28 °C/72 h, pH 5.8), and the mycelium was removed by centrifugation (4500 g, 30 min/4^o^C). The supernatants were treated with absolute Ethanol (3 vol.) to form a precipitate collected by filtration. This residue was solubilized in water, dialyzed (2 kDa cut-off, Spectra-Por®), and freeze-dried to obtain the dried crude LaEPS. For purification, LaEPS was solubilized in water and then submitted to a freeze-thawing treatment (-10^0^ to 20^0^ C) to recover a water-soluble fraction (β-(1→6)-glucan, Linear-LLaEPS). LAEPS (β-(1→3) (1→6)-glucan, Branched-BLaEPS) were recovered by centrifugation (13,000 g, 15 min/5^o^C). This treatment was repeated until no precipitate was observed in fractions LLAEPS, and no more soluble polysaccharides were observed in fraction BLaEPS [6, 8].

### LaEPS carboxymethylation

LaEPS, LLaEPS, or BLaEPS were carboxymethylated according to the protocol described by Kagimura [8]. LaEPS (250 mg) was suspended in 15 mL isopropanol at room temperature and stirred for 15 min. Ten milliliters of 30% NaOH solution (w/v) were slowly added to the mixture and stirred at 50^o^C until the complete solubilization of LaEPS. Subsequently, 3 g of chloroacetic acid (suspended in a small volume of distilled water) was slowly added while stirring. The reaction was refluxed for eight hours at 50^o^C. The mixture was cooled to room temperature and neutralized with glacial acetic acid. The solution was dialyzed against distilled water for eight days with frequent water changes with 12–14 kDa membrane and then freeze-dried to yield LaEPS-C.

### Biological assays

#### Immunomodulatory activity assessment

The LaEPS-C and their fraction’s potential immunomodulatory activity on cytokine secretion were evaluated in human monocyte-derived macrophage cultures. For this purpose, cells were obtained and prepared according to a previously described method [6] following procedures evaluated and approved by the local Research Ethics Committee of the Faculty of Science and Letters of Assis (approval number: CAAE 68135717.6.0000.5401). As suggested by the Committee, written informed consent was obtained from each volunteer before initiating any research procedures. Monocyte-derived macrophages were incubated with different concentrations of LaEPS-C, LLaEPS-C, and BLaEPS-C for 24 and 48 h at 37ºC diluted in RPMI-medium supplemented with 10% FBS for cell viability and cytokine secretion assay. The cell viability was measured using MTT salt, as previously described [38]. Cytokine production was quantified with enzyme immunoassay (ELISA) using commercial kits (BD OptEIA™) following manufacturer instructions. It was assayed with TNF-α, a pro-inflammatory cytokine, and IL-10, an anti-inflammatory. Experimental data were evaluated by analysis of variance (one-way ANOVA), followed by the Bonferroni test. A significance level of P ≤ 0.05 was considered. All assays were in triplicate.

#### In vitro antiproliferative activity assay

Antiproliferative activities for LaEPS, LaEPS-C, LLaEPS-C, and BLaEPS-C were assessed by the sulforhodamine B (SBR) assay [39]. In this assay, different human tumor cell lines were used: MCF-7 (breast), NCI-H460 (lung), NCI ADR/RES (ovary), U-251 (glioma, CNS), 786-0 (kidney), NCI-H460 (lung, non-small cell type), OVCAR-03 (ovarian), HT-29 (colon), K562 (leukemia), PC-3 (prostate) and HaCat (normal cell) which were kindly provided by the National Cancer Institute (NCI). The concentration samples required to produce total growth inhibition or cytostatic effect were determined through non-linear regression analysis using software ORIGIN 8.6® (OriginLab Corporation, Northampton, MA, USA) using the concentration-response curve for each cell line.

#### Antiviral activity

Antiviral activity was evaluated by observing the cell viability of Hep-2 cells (human larynx epidermoid carcinoma cells) in the presence or absence of hRSV (human respiratory syncytial virus). The HEp-2 cell monolayer viability was assessed by colorimetric MTT assays [40]. HEp-2 cells (5 × 10^4^/well) were seeded in 96-well plates and infected with hRSV previously incubated with different LaEPS solutions. Hep-2 cells with untreated RSV or Ribavirin-treated RSV were used as positive and negative infection controls. Cells were maintained in culture conditions (at 37 ºC and 5% CO_2_) for three days when the antiviral activity was correlated with cellular viability (measured using salt MTT). Experimental data were evaluated by analysis of variance (one-way ANOVA), followed by the Bonferroni test. A significance level of P ≤ 0.05 was considered. All assays were in triplicate.

#### Anticoagulant activity: activated partial thromboplastin time (APTT) test and prothrombin time (TP)

The anticoagulant activity was determined using the APTT (Activated Partial Thromboplastin Time) and PT (Prothrombin Time) tests according to the commercial kit (Bios Diagnostica, Sorocaba, São Paulo, Brazil). Different concentrations of LaEPS-C, LLaEPS-C, and BLaEPS-C were incubated (1 min at 37^o^C) with 90 µL plasma (5-100 µg de EPS/mL de plasma). 10 µL Saline solution or heparin (2, 5, and 10 µg) were used as a negative and positive control, respectively. Rabbit cephalin was added to each sample for 3 min. Time coagulation was counted seconds after adding 0.25 M calcium chloride (APTT) or 20 µL thrombin to each sample (PT). The results were expressed as a polysaccharide(µg)/plasma (mL), considering 300 s as the maximum time assay.

### Chemical analysis

#### Water solubility analysis

The methodology to compare the LaEPS and LaEPS-C water solubility was adapted from Wang et al. [41]. 200 mg of both polysaccharides were suspended in 16 mL of distilled water and stirred for 24 h at 25 °C. After centrifugation at 3000 g for 15 min, the supernatants were collected, and the total sugar content of these samples was quantified using the phenol-sulfuric method. So, 0.5 mL of phenol solution 5% (v/v) and 2.5 ml of concentrated sulfuric acid were added to the 2 mL samples of the supernatants. After cooling to room temperature, their absorbance was read in a spectrophotometer at 490 nm. A standard curve was carried out in triplicate with 0.01 to 0.09 mg/mL glucose solution.

#### Elementary Composition Analysis by Energy Dispersive X-Ray detector (EDS)

The concentrations of the sample’s constituent elements were obtained from the Oxford X-ray Energy Dispersive Detector, model INCA-act (Cambridge, United Kingdom), coupled to Carl Zeiss benchtop scanning electron microscope, model EVO LS15 (Jena, Germany). The test used an acceleration voltage of 15.0 kV. Four iterations were completed for the LaEPS.

#### Fourier Transform Infrared (FTIR) and nuclear magnetic resonance (NMR) spectroscopy

FTIR spectra were obtained from a Bruker FTIR spectrometer (Vertex 70, Billerica, EUA) using a KBr disc and ATR (Attenuated Total Reflection). The equipment was operated with a resolution of 4 cm^− 1^, 64 scans, and a scanning range from 4000 to 500 cm^− 1^. ^13^ C NMR spectra were obtained on a Nuclear Magnetic Resonance Imaging Spectrometer (BRUKER, model Bruker Avance III 600 MHz, 14.1T). Deuterium oxide (D_2_O, 99.9%) was used as a solvent for LaEPS and LaEPS-C (10 mg/mL), which were evaluated at 313 K (40 °C). Spectra were recorded using tetramethylsilane as a standard internal reference; chemical shifts (*δ*) were expressed in ppm relative to the ^13^ C signals.

## Results and discussion

*L. theobromae* cultivation yielded 1.7 g of LaEPS. This yield was similar to that of Oliveira et al., which achieved 2.2 g/L of Lasiodiplodan in similar conditions [6]. From an initial mass of 3.7 g of LaEPS and it has purified 1.8 g (44%) of LLaEPS (Linear, soluble, β-(1→6) Lasiodiplodian EPS) and 1.9 g (56%) of BLaEPS (Branched, insoluble, β-(1→3)(1→6) Lasiodiplodian EPS), which was a different result from what was achieved for Oliveira et al. (LLaEPS, 25% and BLaEPS, 75%) [6]. For the LaEPS carboxymethylation, the theoretical mass yield resulted in 1.36 g LaEPS-C/g LaEPS. When comparing the calculated mass (0.23 g/g) and theoretical (1.36 g/g) yield values, it was observed that carboxymethylation represented approximately 17% of the theoretical yield [42–44]. This value is close to the expected value since the carboxymethylation performed by Wang et al. for glucans extracted from *Poria cocos* resulted in a modified polysaccharide mass representing approximately 25% of the theoretical yield [43].

### Biological assays

#### Immunomodulatory Activity Assessment

β-Glucans are well-recognized for their immunomodulatory properties since they stimulate the immune response, initiate inflammatory properties, and promote infection resistance [18]. For instance, (1→3)-β-d-Glucans that have β-d-glucopyranosyl units attached by (1→6) linkages as single unit branches are described as immune system enhancers [45]. β-Glucans bioactivity could be improved by carboxymethylation reaction since it leads to increased water solubility and conformational changes that may affect the basic structure and intermolecular forces [36].

LaEPS and its fractions (1→6)-β-glucan (LLaEPs) and (1→3) (1→6)-β-glucan (BLaEPs) were carboxymethylated to settle if this modification could affect the immunomodulatory activity. So, cell viability analysis was evaluated in human monocyte-derived macrophages and the presence of cytokines: TFN-α (pro-inflammatory) and IL-10 (anti-inflammatory) in the supernatant of cultured monocyte cells.

**Fig. 1:**
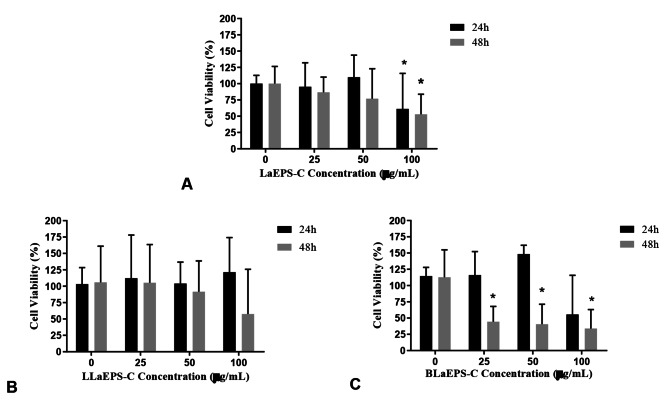
Cell viability analysis by MTT in human monocyte-derived macrophage at four different concentrations and two incubation periods. The 0 (g/mL concentration corresponds to the cell control, and the bars’ values correspond to the means. **(A)** LaEPS-C. **(B)** LLaEPS-C. **(C)** BLaEPS-C

From Fig. 1, both *L. theobromae* EPS (LaEPS-C) and its branched fraction (BLaEPS-C) showed cytotoxicity for human monocyte-derived macrophage. LaEPS-C showed significative toxicity (p < 0.05, lowest viability) only for concentrations above 100 µg/mL in both incubation times: 24 h (61.19 ± 46.97%) and 48 h (52.84 ± 24.06%). Conversely, BLaEPS-C at 48 h, from 25 to 100 µg/mL, has increased significant toxicity (p < 0.05) compared to the control. Non-carboxymethylated BLaEPS and LaEPS toxicity could not be assessed by Olivera et al. because it was water-insoluble [6]. Thus, carboxymethylation increased BLaEPS water solubility and allowed us to evaluate and verify its cytotoxic potential.

LLaEPS carboxymethylation has presented increased water solubility with no significant toxicity in all doses and incubation time tested. Similarly, non-carboxymethylated (1→6)-glucan (LLaEPS) displayed no toxicity toward U937 macrophage cells or PBMC (Peripheral Mononuclear Blood Cells) [6]. Therefore, it can be inferred that the cytotoxicity of LaEPS-C at higher concentrations is due to the presence of BLaEPS-C in its composition (Fig. 1).

**Fig. 2:**
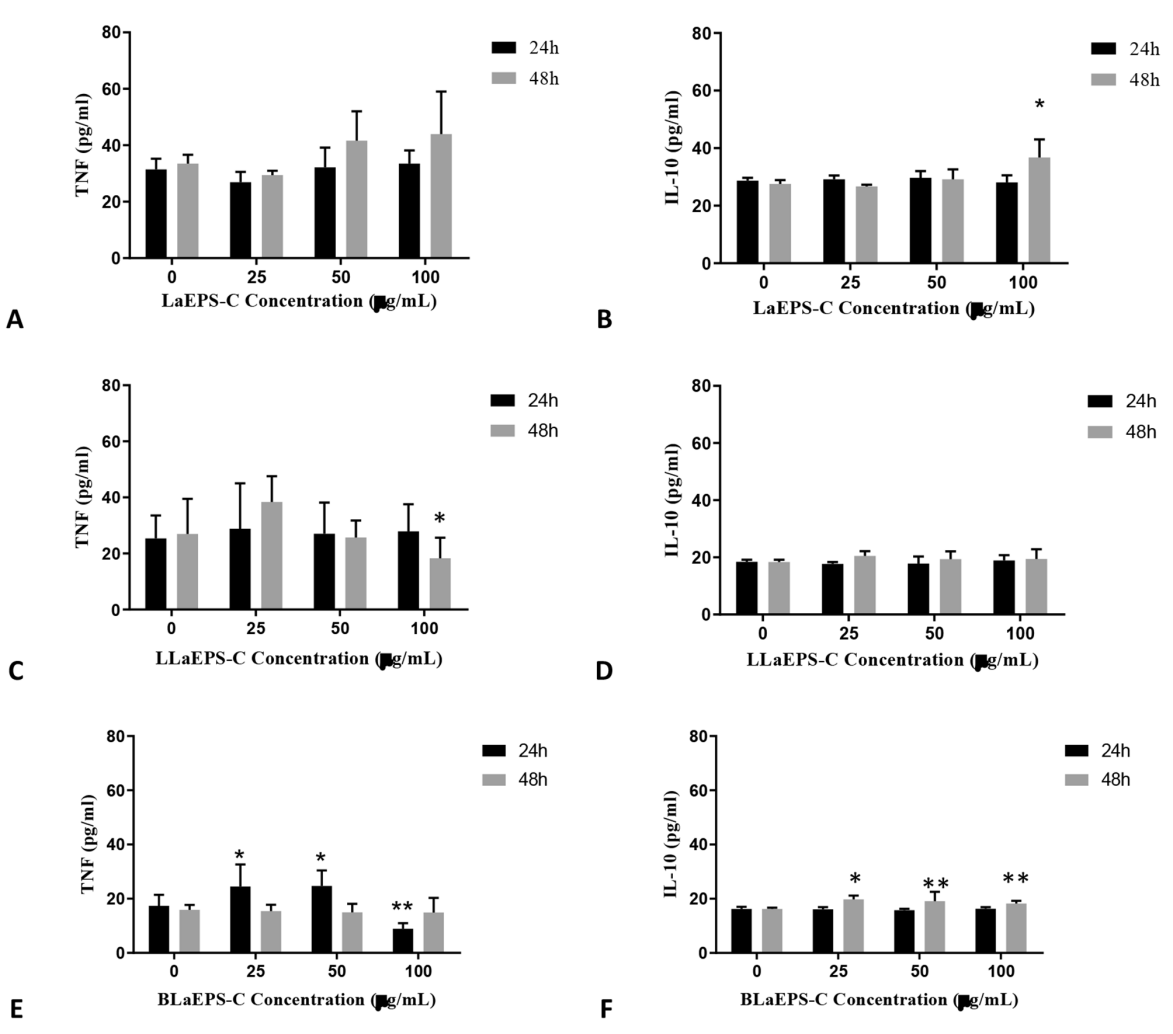
Analysis of the quantification of TNF-α and IL-10 cytokines produced by the culture of human macrophages in pg/mL. **(A)** Production of TNF-α by LaEPS-C. **(B)** Production of IL-10 by LaEPS-C. **(C)** Production of TNF-α by LLaEPS-C. **(D)** Production of IL-10 by LLaEPS-C. **(E)** Production of TNF-α by BlaEPS-C. **(F)** Production of IL-10 by BLaEPS-C

The evaluation of the immunomodulatory activity of LaEPS-C and their fractions stimulating cytokines TFN-α and IL-10 secretion were presented in Fig. 2.

The β-glucans have been described with pro-inflammatory and anti-inflammatory characteristics [6, 38]. From Fig. 2, it could be observed that LaEPS-C presented a light anti-inflammatory character with a significant difference for IL-10 at 100 µg/mL for 48 h (36.75 ± 5.53) (Fig. 2B). For TNF-α cytokine production, LaEPS-C showed no significant difference (p < 0.05) (Fig. 2A).

LLaEPS-C showed a significant decrease in TNF-α production at 100 µg/mL (18.27 ± 5.80, p < 0.05) and 48 h (Fig. 2C). For IL-10 production, this polysaccharide showed no significant difference in time and dosage tested (Fig. 2D). Tests conducted with macrophages showed that the (1→6)-β-glucan (LLaEPS, non-carboxymethylated) induced a modulatory response pattern since the production of TNF-a (a pro-inflammatory cytokine) and IL-10 (an anti-inflammatory cytokine) were increased [6]. So, since LaEPS-C induces IL-10, and LLaEPS-C reduces TNF-α cytokine productions, these polysaccharides could stimulate macrophages to induce an anti-inflammatory activity. Moreover, the carboxymethylation reaction seems to promote a polarization towards the anti-inflammatory profile. Previous work attributes these effects to interactions of linear β-glucans β-(1→3) and/or β-(1→6) with the transmembrane protein Dectin-1 on the surface of macrophages [17, 39].

BLaEPS-C showed induction of TNF-α biogenesis at 24 h, for 25 and 50 µg/mL (24.50 ± 6.15 and 24.69 ± 3.55, respectively), but at 100 µg/mL was observed one opposite effect. At 48 h, there was no significant difference in TNF-α secretion (p < 0.05) (Fig. 2E). For IL-10 synthesis, it was observed a significant difference (p < 0.05) at 48 h in an independent concentration manner (Fig. 2F). One possible explanation is which other cellular receptors are involved in the anti-inflammatory activities of β-(1→3)(1→6) branched β-glucans, including Toll-like receptors 2 (TLR2) and E-series prostanoid receptors 2 and 3 (EP2 and EP3) [46]. However, since cell death can modulate cytokine secretion [47], the immunomodulatory BLaEPS effects on macrophages need to be analyzed carefully because the minimum concentration tested for BLaEPS (25 µg/mL) was cytotoxic to those cells (Fig. 1C).

Together, these results demonstrate that the solubilization provided by polysaccharides carboxymethylation allowed the study of these molecules and the report of different biological effects, including anti- and pro-inflammatory ones. In particular, the anti-inflammatory effects are so interesting since chronic inflammatory responses predispose to a pathological progression of chronic illnesses [48].

#### Evaluation of antiproliferative activities

The anti-tumor activities are closely correlated with the pro-inflammatory profile induced by b-glucans [49]. Their antitumor potency varies on the conformational of their main chain size, branching patterns, and duration of exposure [50]. One possible way to increase b-glucan antitumor activity is *via* carboxymethylation reaction [35].

LaEPS, composed of a branched chain with β-(1→3)(1→6) type bonds plus linear chains with β-(1→6) type bonds, had already been shown in MCF-7 cells (IC_50_ 100 µg/mL) in a time and dose-dependent manner. LaEPS decreases cell proliferation by inducing cell cycle arrest, apoptosis, and oxidative stress [50]. However, Waser says the polysaccharides with the main chain composed of β-(1 → 6) glucan are described with lower activity. Furthermore, β-(1→3) linkages in the main chain of the glucan and additional β-(1→6) branch points are needed for their antitumor action [51].

Compared to LaEPS, LaEPS-C had no activity for MCF-7 cells but showed weak activity for the NCI-ADR/RES cell (Growing Inhibition, GI_50_ 65.3 µg/mL). Although the structure of the main chain of LaEPS-C does not favor the achievement of expressive antitumor activity, these activities could have occurred because of carboxymethylation’s effects on the β-glucan, which could be attributed to the hidrossolubility and structural changes in molecular conformation [23, 51–53]. Conversely, LLaEPS-C did not show any activity for any tumor cell tested, which confirms Wasser’s proposition for low antitumor activity of β-(1 → 6) glucan. BLaEPS-C showed a very weak activity for the K562 cell (GI_50_ of 235 µg/mL). This result was expected since this glucan, produced in a glucose medium, should have lower activity in reducing cell apoptosis and necrosis than in b-glucan produced from a fructose medium [50].

Despite carboxymethylation reaction for LaEPS, LLaEPS, and BLaEPS were not useful for improving antitumor activity, they present no cytotoxicity for HaCat (normal cell, GI_50_ > 250 µg/mL).

In the literature, when these polysaccharides have a main chain composed of β-(1 → 3)-D-glucan and a high proportion of (1 → 6) glucose short-chain structure, they have higher biological activity. Some carboxymethylated β-glucan has been described as having higher in vitro anti-tumoral activity than the non-carboxymethylated β-glucan. Still, despite modifications significantly affecting the primary structure and spatial conformation of β-glucan, some methods show different effects depending on the glucan structures (25,36).

#### Antiviral activity

**Fig. 3:**
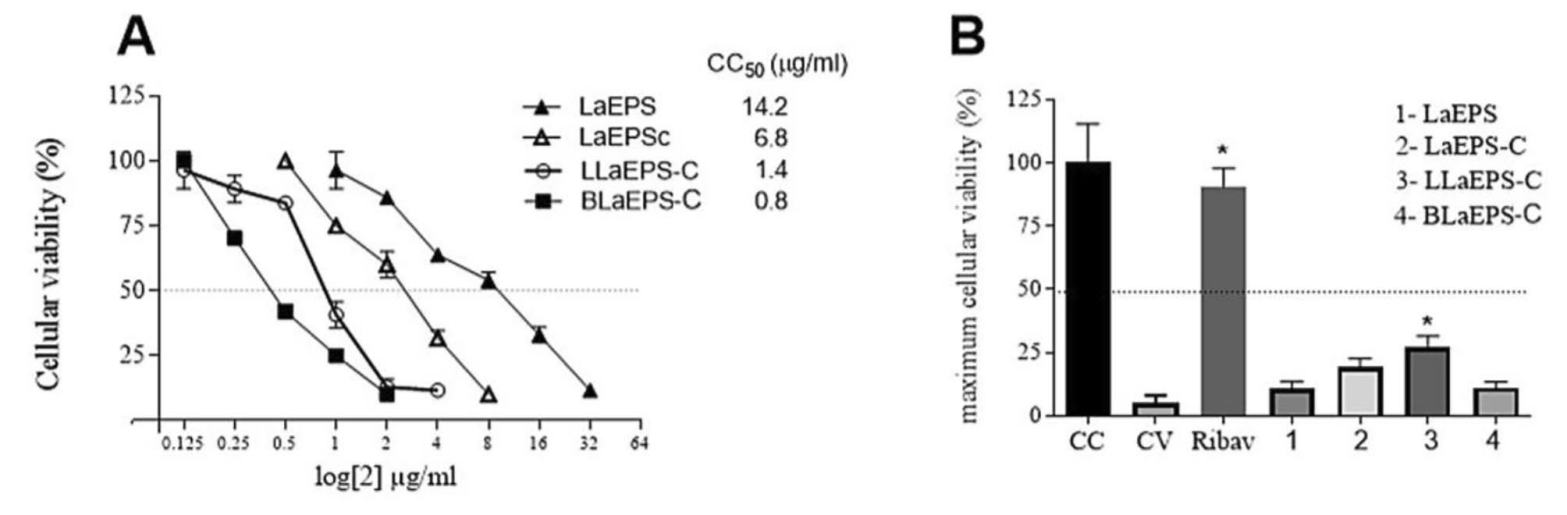
Determination of CC_50_ and anti-hRSV activity. **(A)** Different fractions from EPS were incubated in different concentrations with Hep-2 cells. The cellular viability was evaluated by MTT, and CC_50_ was calculated by linear regression. **(B)** Anti-hRSV from different EPS fractions was tested using a lower CC_50_ concentration than that obtained in Fig. 3A. Just EPS concentration that resulted in the maximum cellular viability is shown in the graphic: LaEPS (7 µg/ml), LaEPS-C (3.4 µg/ml), LLaEPS-C (0.7 µg/ml) and BLaEPS-C (0.4 µg/ml). Cells incubated with culture medium (CC), only virus (CV) or antiviral drug Ribavirin (Ribav) were used as control

The cytotoxicity of molecules with antiviral potential must be previously studied since the benefits of these molecules cannot oppose the tissue damage they can cause. Thus, LaEPS, LaEPS-C, and fractions were tested for viability (cytotoxicity) for Hep-2 cells because this cell is permissive to RSV infection. Cytotoxic activity was determined by CC_50_ (Compound Concentration can kill 50% of the cells in the culture). LaEPS was the least cytotoxic polysaccharide (14.2 µg/ml), followed by LaEPS-C (6.8 µg/ml), LLaEPS-C (1.4 µg/ml), and BLaEPS-C, the most cytotoxic b-glucan (0.8 µg/ml) (Fig. 3). For Hep-2 cells, the solubilization of the LaEPS fractions through the carboxymethylation reaction induced greater cytotoxicity since LaEPS presented the highest CC_50_ value among the tested fractions.

LaEPS-C and fractions had their anti-hRSV (human Respiratory Syncytial Virus) activity tested. For this, were assayed concentrations equal to or smaller than CC_50_ values (Fig. 3). The better anti-hRSV results obtained from each tested LaEPS fraction are shown in Fig. 3. LaEPS (7 µg/ml), LaEPS-C (3.4 µg/ml), and BLaEPS-C (0.4 µg/ml) show discrete anti-hRSV activity that was not statistically significant. LLaEPS-C (0.7 µg/ml) was the only fraction that showed anti-hRSV activity statistically significant, demonstrating that the carboxymethylation improved the antiviral activity from LLaEPS. Moreover, the LLaEPS concentration which was necessary for anti-hRSV activity is lower than toxic concentration to the Hep-2 cells.

The current literature does not present a consensus regarding the advantages or disadvantages of carboxymethylation in the antiviral potential of molecules. Möller et al. reported carboxymethyl groups on hyaluronan derivatives did not contribute to antiviral activity against HSV-1 [54]. Lopes et al. suggest introducing carboxymethyl in polysaccharide groups may change the triple-helix conformation of polysaccharides [9, 30]. Consequently, the hydrophobic interactions responsible for antiviral activity could be damaged [25]. Finally, the negative charges introduced by the carboxymethylation of LaEPS-C and BLaEPS might not be sufficient to confer more extensive hRSV binding. These data from LLaEPS-C are interesting since its antiviral effect occurred at a concentration below its CC_50_; therefore, this glucan has great antiviral potential.

#### Anticoagulant activity: activated partial thromboplastin time (APTT) and prothrombin time (PT) bioassays.

The literature describes anticoagulant effects from sulfated glucans from the lichen *Cladonia ibitipocae* [55]. After sulphation, the β-glucans presented anticoagulant activity similar to heparin [56].

Compared with sulfated β-glucans, carboxymethylated polysaccharides are disadvantaged in this regard. From LaEPS-C and fractions, there was no observed significant difference between positive and negative controls for APTT. So, the inhibition of the clotting may be due to an interaction with different coagulation factors of the common pathway. For instance, the antiplatelet activities of carboxymethylated β-glucans from *Saccharomyces cerevisiae* were only detected for concentrations above 100 µg/mL and antithrombotic activities above 300 µg/mL [57].

Prothrombin, also known as clotting factor II, is a protein produced by the liver and, when activated, promotes the conversion of fibrinogen into fibrin, which, together with platelets, forms a layer that prevents bleeding. Thus, prothrombin is an essential factor for blood clotting to take place. So, Prothrombin Time (PT) is the time of blood clotting begins.

On the other side, unlike sulfated polysaccharides reported above, the carboxymethylated b-glucans showed activity for PT. LaEPS-C revealed a significant reduction in PT when compared to the negative control and heparin (p > 0,05) in a dose-dependent concentration from 50 µg/mL (Table 1). BLaEPS-C showed the same effect only at 50 µg/mL (12.28 ± 1,07s), and LLaEPS-C from 5 µg/mL to 25 µg/mL (11.85 ± 0.87s and 14.26 ± 0.10s).

The anticoagulant effect of heparin is not mediated by modulation of the extrinsic system, but LaEPS-C and fractions seem to be an inhibitor of this pathway [31]. Low steric effects, solubility, and carboxymethyl charge were critical factors in reducing Prothrombin Time (PT) and a procoagulant effect of LLaEPS-C. Above 50 µg/mL LLaEPS, as viscosity grows, there is an increase in Prothrombin Time (PT) and a reduction in the procoagulant effect. For BLAEPS-C, it could be deduced a higher steric effect is responsible for low activity. Conversely, LaEPS-C reduces its PT due to the LLaEPS-C in its composition.

**Table 1:** Assessment of anticoagulant activity by APTT and PT.

Sample	Concentration (µg/mL)	APTT (s)	PT (s)
Negative Control*		38.59 ± 0.60 a	15.04 ± 0.49 a
**Heparin**	10	34.31 ± 3.38 a	14.05 ± 1.30 a
	25	130.36 ± 4.85 bc	20.69 ± 0.77 bc
	50	> 300 bcd	24.76 ± 0.98 bcd
	100	> 300 bcde	29.68 ± 0.94 bcde
**LaEPS-C**	5	36.87 ± 2.79 a	14.90 ± 0.64 a
	10	36.24 ± 0.74 a	14.65 ± 0.90 a
	25	35.59 ± 1.50 a	12.95 ± 0.17 a
	50	31.29 ± 0.23 a	11.73 ± 0.24 b
	100	31.75 ± 0.86 a	10.73 ± 0.63 b
**BLaEPS-C**	5	38.91 ± 0.45 a	15,73 ± 0,26 a
	10	41.13 ± 1,38 a	14.98 ± 0,85 a
	25	32.29 ± 4,98 a	14.56 ± 1,14 a
	50	32.32 ± 0,65 a	12,28 ± 1,07 b
	100	33.55 ± 4.02 a	13.38 ± 1.86 a
**LLaEPS-C**	5	44.21 ± 1.92 a	12.63 ± 0.28 b
	10	37.56 ± 1.35 a	11.85 ± 0.87 b
	25	34.69 ± 1.61 a	14.26 ± 0.10 b
	50	35.31 ± 1.82 a	12.51 ± 0.66 a
	100	32.76 ± 0.34 a	13.43 ± 1.24 a

*Blood plasma with saline only (no LaEPS or heparin added)** Clotting time values ​​followed by the same letters do not differ statistically from each other (p > 0.05). The reference values for normality are 30–45 s and 10–14 s for the APTT and PT, respectively

#### Chemical analysis

The LaEPS-C had 3.4 times more soluble sugar (0.68 mg/mL H_2_O) than LaEPS (0.2 mg/mL H_2_O). Interactions of hydroxyl groups in LaEPS decrease after carboxymethylation, producing the molecule more hydrophilic [7, 8]. So, the LaEPS carboxymethylation could be associated with changes in the solubility in water [23].

In LaEPS-C and LaEPS Elementary Composition Analysis by Energy Dispersive X-Ray Detector (EDS) was observed an increase in oxygen (43.95–45.08%) and carbon decrease (55.59–52.71%) proportions. These results were related to substituting a hydroxyl (OH) of the glucose ring with a carboxymethyl radical (-CH_2_COOH). The percentage of silica (0.24%) resulted from the fungus culture. Na (1.12%) remaining in the LaEPS-C sample is a residue of the carboxymethylation reaction. Also, LaEPS and LaEPS-C detected magnesium (0.13 and 0.23%) and calcium (0.11 and 0.43%) from the fungus culture. The Au percentages in the LaEPS and LaEPS-C (0.22%, 0.19%) correspond to the insertion of the element in the LaEPS metallization procedure under vacuum, so the analysis by Electron Microscopy of Scan could be performed should be disregarded.

LaEPS and LaEPS-C FTIR spectrum analysis of the absorption bands assigned revealed one typical polymeric structure of the carbohydrate. In the LaEPS-C spectra, two new intermediate absorption bands were observed, in 1604 cm^− 1^ and 1421 cm^− 1^, respectively, from stretching the asymmetric and symmetrical COO^−^. These bands indicate the occurrence of carboxymethylation of the polysaccharide. A weak absorption band at 1720 cm^− 1^ belonging to carboxyl groups suggests that the LaEPS-C sample exists predominantly as a salt [42]. The strong absorption band between 3310 cm^− 1^ and 3425 cm^− 1^ for both spectra is attributed to OH stretching vibrations. The peak at 2920 cm^− 1^ was attributed to the CH stretching of the methylene groups (CH_2_). The peak at 1648 cm^− 1^ found in the LaEPS spectrum was attributed to the glucose ring. At 1604 cm^− 1^, a higher peak in the LaEPS-C sample can be attributed to the glucose ring associated with the absorption band corresponding to COO^−^ [35].

The characteristic signals of LAEPS-C were confirmed through chemical shifts of ^13^ C NMR and comparison with literature data [7, 8]. Among them, δ 178.1 (carbonyl of the carbomethoxy group) and δ 70.0 were attributed to methylene of the carbomethoxy group. Shift signals for C3 (76.3 to 85.1 ppm) and C4 (71.0 to 79.6 ppm) suggest that this carboxymethylation may have occurred at carbons 3 and 4. The peaks at 102.8 were attributed to glucopyranose units (C-1), 73.3 (C-2), 60.9 (C-5), and 71.2 (C-6) [35, 52].

## Conclusions

The carboxymethylation of LaEPS (Linear plus Branched), LLaEPS (Linear), and BLaEPS (Branched) increased water solubility because of changes in the charge and conformation. Also, the steric effects could be better established for these polysaccharides, and their anti-inflammatory, anti-hRSV, and anticoagulant activities could be tested.

LaEPS-C and LLaEPS-C have anti-inflammatory activity with very low cytotoxicity to the macrophages. Compared to them, BLaEPS has an anti-inflammatory effect, with improved cytotoxicity, because of their higher steric effects.

LaEPS-C showed low antiproliferative activity against NCI-ADR/RES, and BLaEPS-C presented slight activity for K562. LLaEPS-C did not have activity for all tested cells. For HaCat, a normal cell, these carboxymethylated glucans showed no activity. So, we suggest LLaEPS-C reduced steric effects seemed to be a critical factor in the lack of antiproliferative activity. Still, LLaEPS-C effectively enhanced anti-hRSV, with low cytotoxicity in Hep-2 cells. For BLaEPS and LaEPS, it was observed no antiviral activity.

The anticoagulant activity of the carboxymethylated compounds was significantly enhanced. LaEPS-C and fractions promote a reduction in PT (procoagulant effect). LLaEPS-C showed better PT activity, especially at low concentrations. As LLaEPS-C concentration increases, the interaction between LLaEPS-C-fibrinogen decreases because of the increase in viscosity. LaEPS-C and BLaEPS-C, with higher sterical than LLaEPS-C effects, do not suffer the effects of increased viscosity and become more effective for higher concentrations.

From the results above, Carboxymethylated Linear *Lasiodiplodia theobromae* β-glucan (LLaEPS-C) with low steric effects stands out in biological assays from those carboxymethylated b-glucans tested. For this reason, further assessment of the LLaEPS-C structure-activity relationship studies is required to detect new and desirable bioactivities from this polysaccharide.

## Data Availability

The datasets used in the current study are available from the corresponding author on reasonable request.

## List of abbreviations

LaEPS: *Lasiodiplodia theobromae* β-glucan exopolysaccharide
LaEPS-C: Carboxymethylated *Lasiodiplodia theobromae* β-glucan exopolysaccharide
LLaEPS: Linear *Lasiodiplodia theobromae* β-glucan exopolysaccharide
LaEPS-C: Carboxymethylated Linear *Lasiodiplodia theobromae* β-glucan exopolysaccharide
BLaEPS: Branched *Lasiodiplodia theobromae* β-glucan exopolysaccharide
BLaEPS-C: Carboxymethylated Branched *Lasiodiplodia theobromae* β-glucan exopolysaccharide
EDS: Elementary Composition Analysis by Energy Dispersive X-Ray Detector
FTIR: Fourier Transform Infrared
NMR: Nuclear Magnetic Resonance
APTT: Activated Partial Thromboplastin Time
PT: Prothrombin Time
IL-10: Interleukin (IL)-10
TNF-α: Tumor necrosis factor alpha
hRSV: Human respiratory syncytial virus
MTT: 3-(4,5-dimethyl-2-thiazolyl)-2,5-diphenyl-2-H-tetrazolium bromide
